# Embroidered Copper Microwire Current Collector for Improved Cycling Performance of Silicon Anodes in Lithium-Ion Batteries

**DOI:** 10.1038/s41598-017-13261-y

**Published:** 2017-10-12

**Authors:** Ben Breitung, Noemí Aguiló-Aguayo, Thomas Bechtold, Horst Hahn, Jürgen Janek, Torsten Brezesinski

**Affiliations:** 1 Battery and Electrochemistry Laboratory, Institute of Nanotechnology, Karlsruhe Institute of Technology, Hermann-von-Helmholtz-Platz 1, 76344 Eggenstein-Leopoldshafen, Germany; 2 Research Institute of Textile Chemistry and Textile Physics, Leopold-Franzens-University Innsbruck, Höchsterstraße 73, 6850 Dornbirn, Austria; 3 Helmholtz Institute Ulm for Electrochemical Energy Storage, Helmholtzstraße 11, 89081 Ulm, Germany; 4 Institute of Physical Chemistry, Justus-Liebig-University Giessen, Heinrich-Buff-Ring 17, 35392 Giessen, Germany

## Abstract

Si holds great promise as an alloying anode material for Li-ion batteries with improved energy density because of its high theoretical specific capacity and favorable operation voltage range. However, the large volume expansion of Si during electrochemical reaction with Li and the associated adverse effects strongly limit its prospect for application. Here, we report on the use of three-dimensional instead of flat current collectors for high-capacity Si anodes in an attempt to mitigate the loss of electrical contact of active electrode regions as a result of structural disintegration with cycling. The current collectors were produced by technical embroidery and consist of interconnected Cu wires of diameter <150 µm. In comparison to Si/Li cells using a conventional Cu foil current collector, the embroidered microwire network-based cells show much enhanced capacity and reversibility due to a higher degree of tolerance to cycling.

## Introduction

At present, lithium-ion batteries (LIBs) represent the preferred and most reliable energy storage devices for both electric vehicles and consumer electronics^1–3^. The cathode of state-of-the-art LIBs usually contains one or more tailored active materials, with the most common being LiFePO_4_ (LFP), LiMn_2_O_4_ (LMO), LiCoO_2_ (LCO), LiNi_*x*_Co_*y*_Mn_*z*_O_2_ (NCM), and LiNi_0.8_Co_0.15_Al_0.05_O_2_ (NCA)^4–7^. The anode material of choice is graphite, showing good reversibility and longevity (i.e., cycle and calendar life) in cells with carbonate-based electrolyte at reasonable specific capacities. The lack of diversity in practical negative electrode materials stems from the relatively small number of viable alternatives with similar performance metrics in terms of cyclability, including initial capacity loss, capacity retention, voltage efficiency etc. Yet, particularly complex anode structures are receiving much attention in recent years, and more studies in this area might help to pave the way for the development of a new generation of LIBs with improved gravimetric and volumetric energy densities^8^.

There are different kinds of negative electrode materials, which can be classified based on the reaction type into: intercalation/insertion, conversion and alloying materials^8–15^. While graphite is the most prominent example of an intercalation material, some transition metal oxides and non-oxides as well as certain elements are capable of undergoing conversion and/or alloying reactions with Li^8,11–13^. Silicon (Si) is one such alloying type anode material that holds great promise for LIB applications^16–19^. Its theoretical specific capacity is as high as ~4200 mA h g^−1^ when fully lithiated to a composition of Li_22_Si_5_ or ~3580 mA h g^−1^ for Li_15_Si_4_. These storage capacities are an order of magnitude higher than for graphite anodes (roughly the same applies to volumetric capacities). However, the oxophilic nature of Si and other fundamental issues related to the electrochemical reaction with Li strongly limit its application possibilities. Major issues of Si-based electrodes arise from large volume changes during operation (>300% difference between lithiated and delithiated states)^20^. This continuous swelling and shrinking causes mechanical stress, leading to material fracture and pulverization and eventually giving rise to a number of undesired side reactions such as increased electrolyte decomposition^21–24^. Thus, small-size particles are often used in an attempt to somewhat counteract these adverse effects and to achieve fast kinetics or, in other words, short solid-state diffusion lengths for efficient charge transfer^18,25,26^. The reasons are that nanoscale Si, particularly when in amorphous form, is known to be able to withstand stress-induced cracking to a higher degree than microcrystalline material and that pure Si is an insulator^27–29^. However, the loss of electrical contact of active electrode regions as a result of crack formation and delamination from the current collector with cycling remains one of the major challenges of Si anodes.

## Results

Here, we report on the use of 3D networks of copper (Cu) microwires in place of conventional foil as current collector for Si-based electrodes to address the above issues. Current collectors with different spacings between the wires (i.e., different wire densities), and thus also varying accessible surface areas, were fabricated by technical embroidery. The general idea was to have a conductive and robust network that pervades the entire electrode volume, thereby (i) better accommodating the volume changes of Si, (ii) ensuring good electrical connectivity between the current collector and the Si/carbon black/polymer binder composite electrode and (iii) reducing the average diffusion distance. Ultimately, this should lead to improvements in cycling performance and stability.

**Figure 1:**
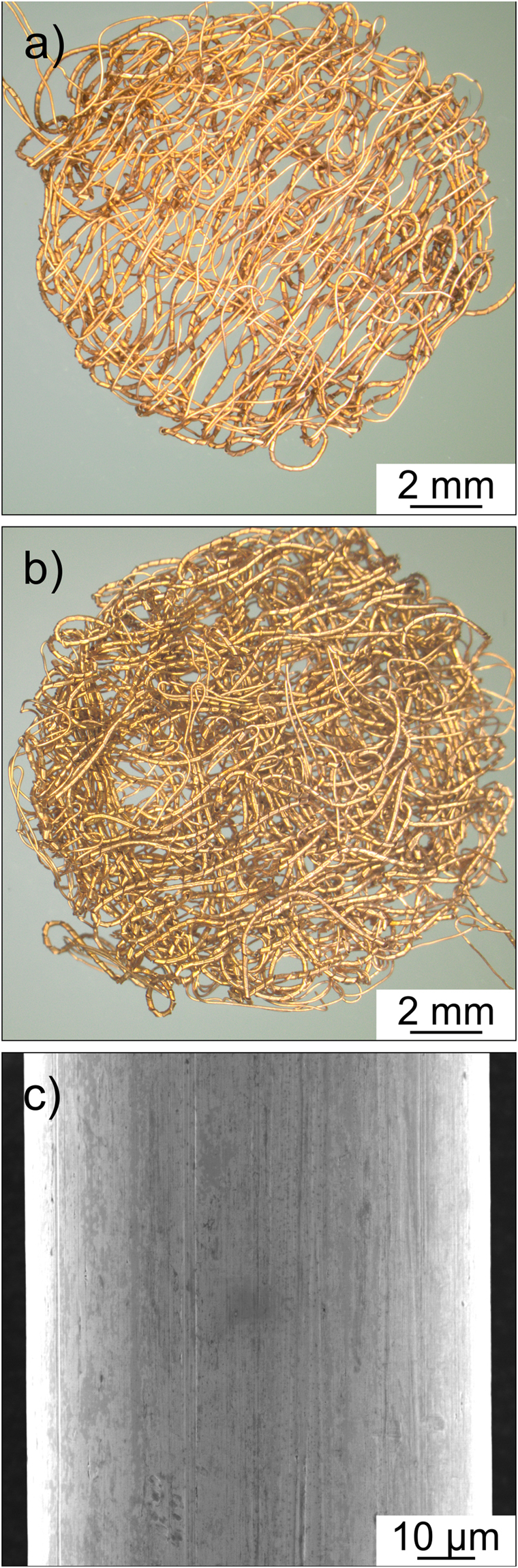
(**a**,**b**) Photomicrographs of embroidered Cu current collectors with different wire densities. (**c**) SEM image of the surface of a single microwire.

Panels (a) and (b) of Fig. 1 present photomicrographs of two different embroidered current collector samples, of which only the one shown in (a) was employed in this work. The embroidery process, in principle, allows for the preparation of a wide variety of materials of various sizes, shapes and structures. It involved the use of two yarns, so-called front and back yarns. The current collectors were embroidered on polyester (PES) fabric using 80 µm-diameter Cu wire as front yarn. Cu/PES wrapped yarn of diameter around 150 µm was utilized as back yarn, from which the PES could be removed readily by base leaching. The current collectors were fabricated in the form of 13 mm circular discs to be used directly in coin cells. Their nominal thickness without compression was around 0.45 mm. The accessible surface area of the current collector in panel (a) was calculated to be in range between 3.6 cm^2^ and 4 cm^2^. This is about three times the geometrical area of flat Cu foil of the same size. The SEM images at different magnifications in panel (c) and Fig. S1 (Supplementary Information) indicate that the wire surface is relatively smooth, without notable features on the micrometer and submicrometer scales (besides few traces of the manufacturing process). According to XRD (see Fig. S2 in the Supplementary Information), the wires consist of cubic Cu crystallites of space group *Fm* $\mathop{3}\limits^{\bar{} }$ *m*, and the presence of larger amounts of impurity phases such as Cu_2_O can be ruled out.

**Figure 2:**
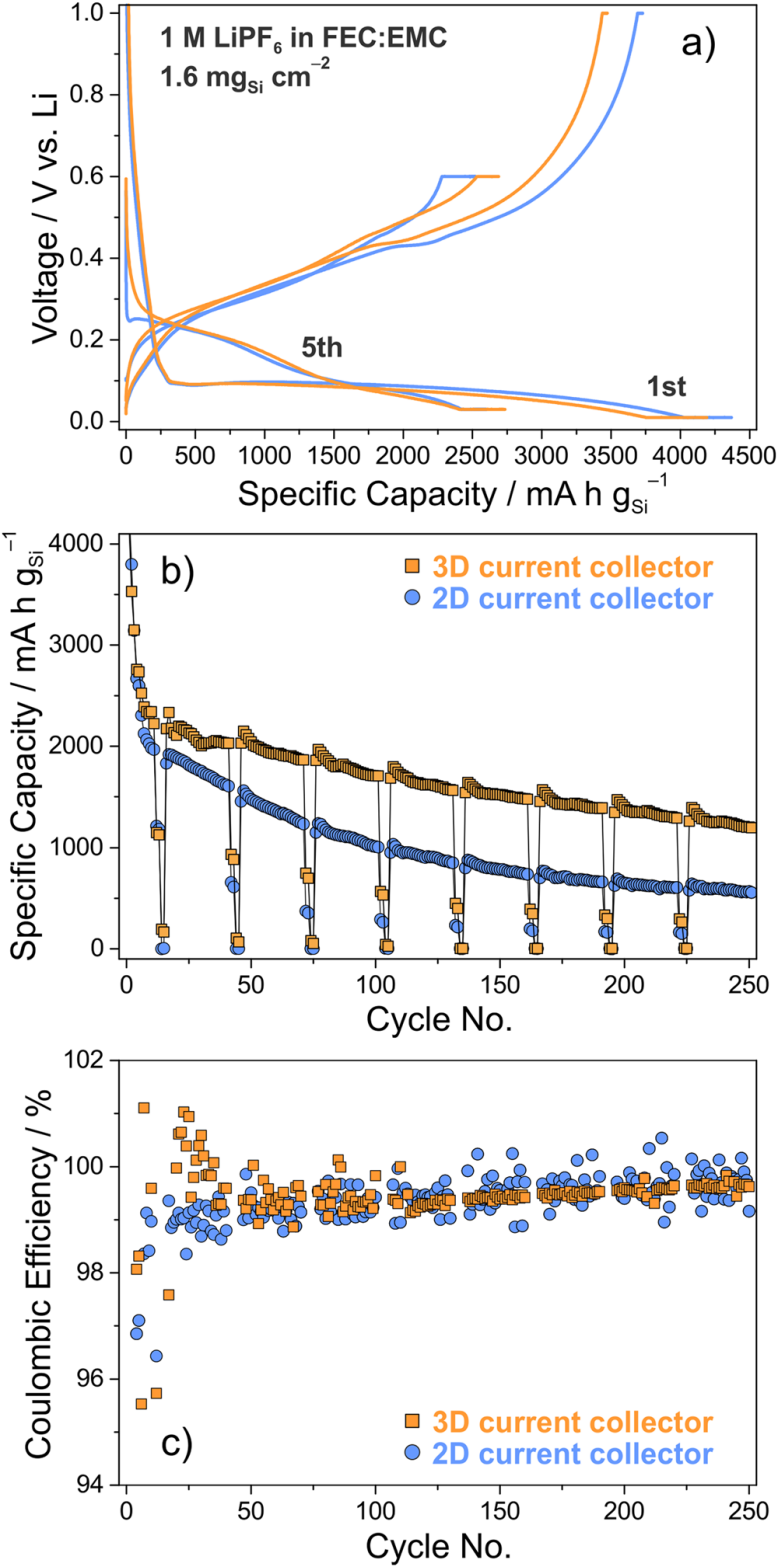
Long-term cycling performance of Si-based electrodes with 2D and 3D current collectors. After the first two activation cycles at C/20 in the voltage range from 1000 mV to 10 mV were completed, the cells were cycled between 600 mV and 30 mV for the subsequently cycles, the first three of which were performed at C/10. Then, the C-rate was set to C/2 charge (alloying), while that on discharge (dealloying) was varied from C/10 to 3 C. A constant voltage step at the cutoff voltages was applied until the current dropped below C/20, except during rate performance testing. (**a**) Voltage profiles of the initial and fifth cycle at C/20 and C/10, respectively. (**b**) Capacity retention and (**c**) Coulombic efficiency versus the cycle number.

The electrochemical testing was performed at 25 °C in coin cells with a lithium metal counter electrode. The Si loading was adjusted to around 1.6 mg cm^−2^, which should allow for areal capacities similar to commercial LIBs, and the same volume of FEC:EMC-based electrolyte was used in all cells. The cyclability between 600 mV and 30 mV was evaluated at a charging (Li insertion or alloying) rate of C/2; the C-rate on discharge (Li extraction or dealloying) was varied from C/10 to 3 C. However, the first five cycles, which can be referred as activation or formation cycles, were performed at lower rates of C/20 or C/10. The cutoff voltages were set at 1000 mV and 10 mV in the initial two cycles to ensure that the bulk of the electrodes is electrochemically addressed to the greatest extent possible (see Tab. S1 in the Supplementary Information for details on the cycling protocol). Fig. 2 shows the cycling performance of Si/Li cells using 2D and 3D current collectors. Note that 2D refers to Cu foil, while 3D refers to the embroidered microwire network. Representative charge-discharge curves are depicted in panel (a). As can be seen, the electrochemical alloying of Si and Li occurs at about 100 mV in the first cycle at C/20. The capacity delivered before the active material converts into Li_*x*_Si species is due to solid electrolyte interface (SEI) formation on the free surface of both the Si particles and carbon black additive and likely other irreversible side reactions. The lithiation capacities achieved with both the 2D and 3D current collectors are in the range of 4200–4350 mA h g^−1^ in the first cycle. The initial Coulombic efficiencies are also similar at around 84%. Likewise, the delithiation profiles show the same general shape, with a subtle plateau at about 430 mV. The appearance of this plateau is indicative of the formation of Li_15_Si_4_ with lithiation and has been shown to be dependent on the Si material used and its interaction with the polymer binder^19^. From the second cycle onward, the onset of lithiation is shifted towards higher voltages irrespective of the C-rate. The primary reasons are the amorphization during the initial alloying cycle and changes in electrode structure and composition.

The capacity retention is depicted in panel (b). The electrodes experience a significant decrease in specific capacity within the first ten cycles. Besides the destruction and reformation of the SEI (and growth with cycling) and other parasitic reactions such as lithium consumption due to conversion of the native oxide layer on the Si particles^30^, this is due to the fact that the lower and upper cutoff voltages were increased and decreased, respectively, for stability reasons. As anticipated, cells using the 3D current collector indeed deliver higher specific capacities and exhibit much improved stability and rate capability. In contrast to the exponential type decay observed for the electrodes on Cu foil, the specific capacity decreases in a nearly linear fashion from 2200 mA h g^−1^ or 3.5 mA h cm^−2^ (10th cycle) to 1200 mA h g^−1^ or 1.9 mA h cm^−2^ (250th cycle), corresponding to an average fade rate per cycle of 0.19%. We note that already after twenty-five cycles the Si-based electrodes on Cu foil show a considerably higher overvoltage for the electrochemical reaction with Li (see Fig. S3 in the Supplementary Information). This finding helps to explain the improvement in cycling performance achieved when using the embroidered current collector. The same is also seen in the later cycles and for higher C-rates (see charge-discharge curves in Fig. S4 in the Supplementary Information). Similar behavior was recently observed by two of the authors for LFP-based cathodes with embroidered Al microwire current collector, also exhibiting lower overvoltage upon charge and discharge than the planar configuration due to lower charge transfer resistance^31^. Overall, the “non-conventional” electrodes are capable of delivering about twice the specific capacity at C/2 after 250 cycles. In addition, they show better rate capability (kinetics), as mentioned above. However, from the data in panel (b), it is clear that the Si anodes employed here are little suited for power applications unless they are optimized.

The same trend in terms of stability can be seen from the Coulombic efficiency data in panel (c). The capacity loss in the initial cycle is around 16%. Yet, within about ten cycles the Coulombic efficiency stabilizes above 99%. Notably, it is greater than 99.5% after 200 cycles, which is relatively high for Li cells using non-optimized high-capacity Si-based electrodes. Also, as is evident, there is less scatter in the data for the 3D current collector-based cells, particularly at higher cycle numbers. This is indicative of less degradation or, in other words, better cycling tolerance, thus providing indirect evidence for the positive (stabilizing) effect that the embroidered Cu microwire current collector has on the electrochemical performance.

**Figure 3:**
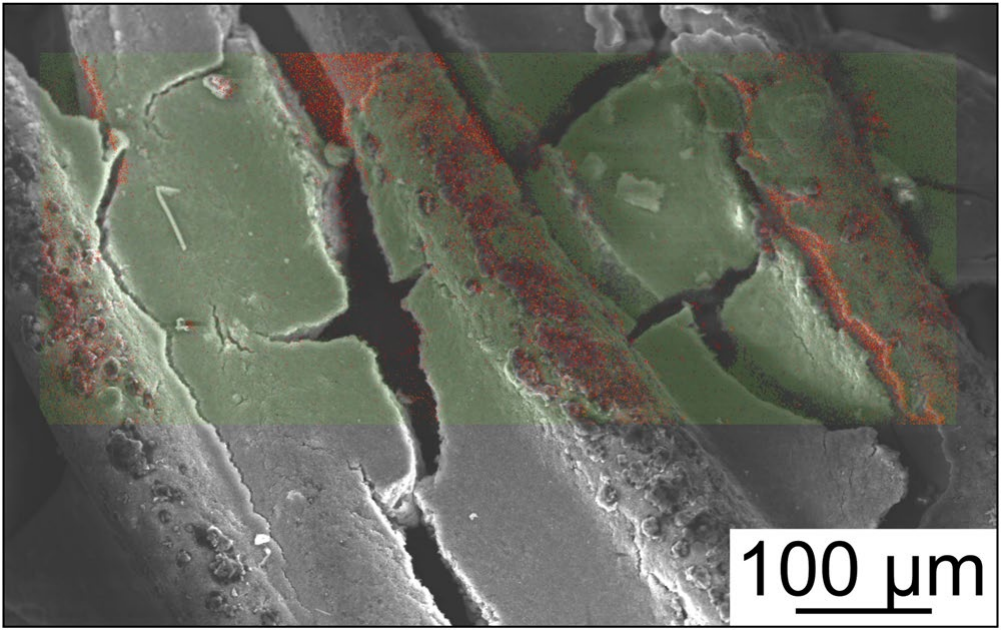
Top view SEM image of a cycled Si-based electrode with 3D current collector and the corresponding EDX map showing the distribution of Si in green and Cu in red.

Collectively, the data in Figs. 2, S3 and S4 demonstrate that both the active material utilization and the capacity retention can be significantly improved by replacing Cu foil with a 3D current collector that is directly embedded into the structure of the electrode^32,33^. In so doing, it seems that better electrical connectivity between the current collector and the electrode is maintained over many cycles. However, more importantly, the material displacement during operation, and with that detachment of active electrode regions from the current collector, can be mitigated to a larger extent. This is corroborated by the finding that Si-based electrodes with 3D current collector also exhibit micrometer-sized cracks after cycling (see SEM image and EDX mapping analysis in Fig. 3). Nevertheless, apparently, the different electrode regions remain electrically connected, and thus electrochemically active, unlike in the case of electrodes on flat Cu foil. Besides, the imaging and mapping results suggest a strong interaction (high affinity) between the embroidered current collector and the carbon black/polymer binder composite, with the latter probably serving as interface between the Cu wires and the Si particles (see Fig. S5 in the Supplementary Information); yet, the detailed relationship among Cu, C, polymer binder and Si needs further study.

## Discussion

We believe that 3D micro- and/or nanoscale current collectors might become viable alternatives to flat ones if their volumetric density and structure can be tailored to specific needs while maintaining mechanical robustness and the processing can be made cost-efficient. Overall, they appear particularly suited when the areal loading is a major concern or electrode breathing is an issue, as is the case with the vast majority of the conversion and alloying type electrode materials. Besides, the embroidered current collectors can be produced in different shapes and sizes, thereby increasing their versatility and enabling them to compete with conventional current collectors.

## Methods

### Materials

Water-based slurry was prepared by blending 63 wt.% Si particles of size <100 nm, (≥98%, Sigma-Aldrich), 22 wt.% Super C65 carbon black additive (Timcal) and 15 wt.% poly(vinyl alcohol) Selvol 425 binder (Sekisui). Electrodes were obtained by casting the slurry either on 18 µm-thick Cu foil (Gould Electronics) or in embroidered Cu microwire networks as current collectors. In the latter case, excess material was wiped off to ensure good contact between the different components in the cell (see Fig. S6 in the Supplementary Information for more details). Finally, the electrodes were dried at 80 °C in vacuum for 12 h. The areal loading was adjusted to around 1.6 mg_Si_ cm^−2^.

### Electrochemical testing and instrumentation

Coin type cells for long-term cycling tests were assembled inside an Ar-filled glovebox (MBraun) by stacking 600 µm-thick Li metal foil (Rockwood Lithium Inc.), glass microfiber film separator of GF/D type (GE Healthcare Life Sciences, Whatman) soaked with electrolyte solution and Si-based electrode. 1 M LiPF_6_ in a 1:1 (w/w) mixture of fluoroethylene carbonate (FEC, Solvay) and ethyl methyl carbonate (EMC, BASF SE) was used as electrolyte. Electrochemical testing was performed at 25 °C and at rates ranging from C/10 to 3 C, with 1 C = 4008 mA g_Si_^−1^, using a MACCOR Series 4000 cycler (Tulsa). X-ray diffraction (XRD) was performed on a Bruker AXS D8 Advance equipped with a LynxEye silicon strip detector (*λ* = 0.15418 nm). Scanning electron microscopy (SEM) and energy-dispersive X-ray spectroscopy (EDX) were performed using both LEO 1530 and JEOL JSM-7100F microscopes operated at 10–20 keV.

## Electronic supplementary material

Supplementary Information
