# Supplementary material for: Embroidered Copper Microwire Current Collector for Improved Cycling Performance of Silicon Anodes in Lithium-Ion Batteries
Source: Sci Rep. 2017 Oct 12;7:13010. doi: 10.1038/s41598-017-13261-y (PMC5638807; doi:10.1038/s41598-017-13261-y)
Supplement: Supplementary file 1 — Supplementary Information [file 41598_2017_13261_MOESM1_ESM.pdf]

Embroidered Copper Microwire Current Collector for Improved Cycling Performance of Silicon Anodes in Lithium-Ion Batteries

Ben Breitung[1], Noemí Aguiló-Aguayo[2], Thomas Bechtold[2], Horst Hahn[1,3], Jürgen Janek[1,4] & Torsten Brezesinski[1]

[1]Battery and Electrochemistry Laboratory, Institute of Nanotechnology, Karlsruhe Institute of Technology, Hermann-von-Helmholtz-Platz 1, 76344 Eggenstein-Leopoldshafen, Germany

[2]Research Institute of Textile Chemistry and Textile Physics, Leopold-Franzens-University Innsbruck, Höchsterstraße 73, 6850 Dornbirn, Austria

[3]Helmholtz Institute Ulm for Electrochemical Energy Storage, Helmholtzstraße 11, 89081 Ulm, Germany

[4]Institute of Physical Chemistry, Justus-Liebig-University Giessen, Heinrich-Buff-Ring 17, 35392 Giessen, Germany

Correspondence and requests for materials should be addressed to B.B. (email: ben.breitung@kit.edu) or T.Br. (email: torsten.brezesinski@kit.edu)

Table S1. Detailed cycling protocol used for testing of Si/Li cells.

Step	Process	Cutoff Voltage	C-Rate	Mode	Number of Cycles
1	Lithiation	10 mV	C/20	CC/CV	2
2	Delithiation	1000 mV	C/20	CC/CV	
3	Lithiation	30 mV	C/10	CC/CV	3
4	Delithiation	600 mV	C/10	CC/CV	
5	Lithiation	30 mV	C/2	CC/CV	5
6	Delithiation	600 mV	C/2	CC/CV	
7	Lithiation	30 mV	C/2	CC/CV	2
8	Delithiation	600 mV	1C	CC	
9	Lithiation	30 mV	C/2	CC/CV	2
10	Delithiation	600 mV	3C	CC	
11	Lithiation	30 mV	C/2	CC/CV	1
12	Delithiation	600 mV	C/10	CC	
13	Lithiation	30 mV	C/2	CC/CV	25
14	Delithiation	600 mV	C/2	CC/CV	

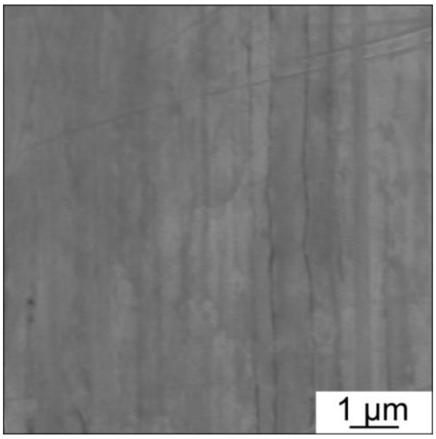

Figure S1. High-magnification SEM image of the surface of a single Cu microwire. There is no significant roughness, nor any notable features on the micrometer level.

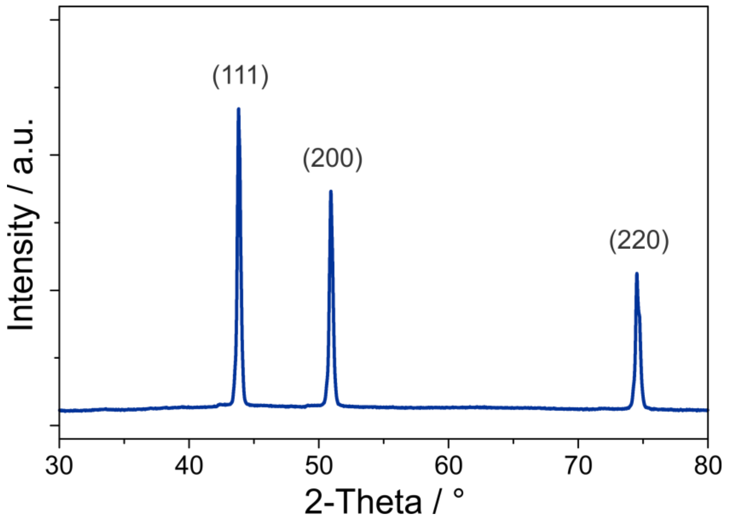

Figure S2. XRD pattern obtained on a 3D current collector. The microwires consist of cubic Cu crystallites (space group $Fm\bar{3}m$, no. 225) with an average size of 30 nm (Scherrer analysis). The amount of crystalline impurity phases is below 5%.

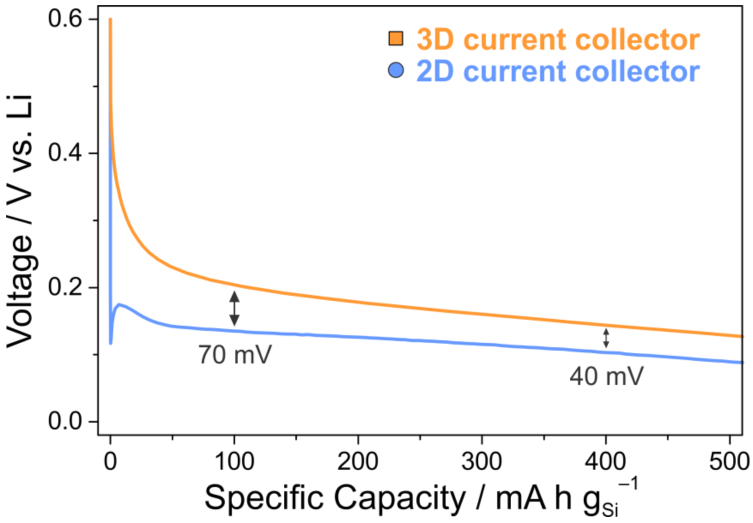

Figure S3. Lithiation profiles (25th cycle at C/2) of Si-based electrodes with 2D and 3D current collectors. Electrodes on conventional Cu foil exhibit higher overvoltage (several tens of mV) for the electrochemical alloying reaction with Li. This result helps to explain the improvement in both capacity and cycling stability achieved when using the embroidered Cu microwire current collector.

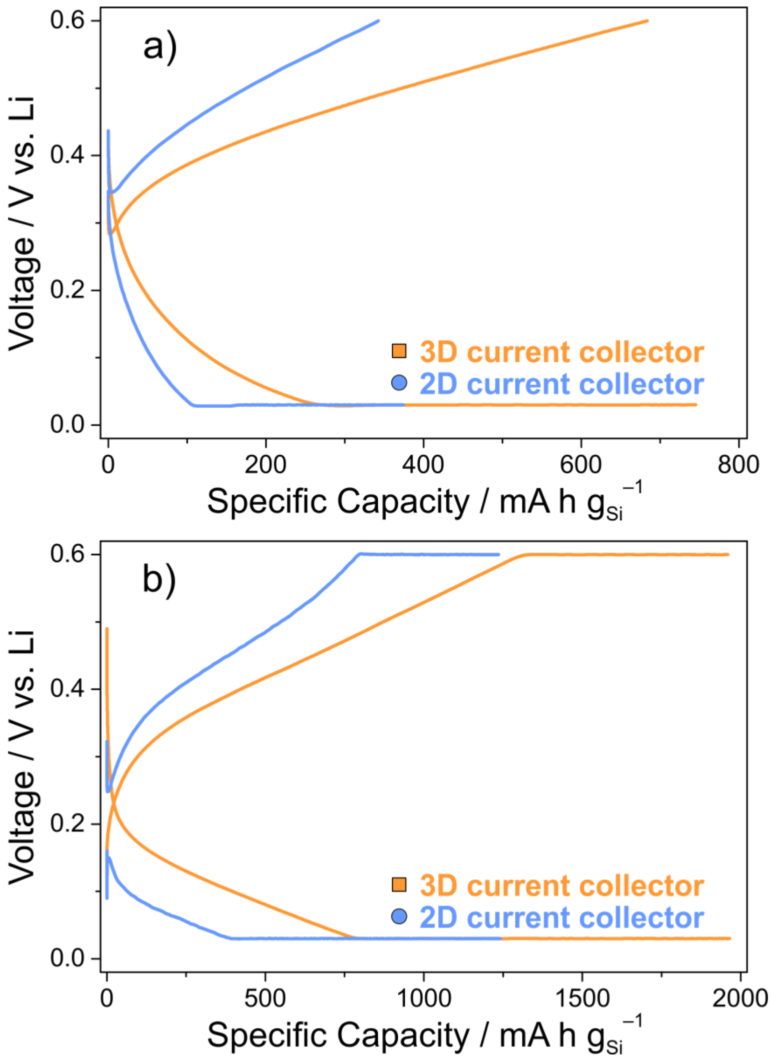

Figure S4. Voltage profiles of Si-based electrodes with 2D and 3D current collectors. (a) 72nd cycle at C/2 charge (lithiation; CC/CV mode) and 1C discharge (delithiation; CC mode). (b) 77th cycle at C/2 charge (lithiation; CC/CV mode) and C/2 discharge (delithiation; CC/CV mode). As is evident, Si/Li cells using the embroidered Cu microwire current collector exhibit much lower overvoltage and better kinetics, thus leading to higher specific capacities and improved cycling stability.

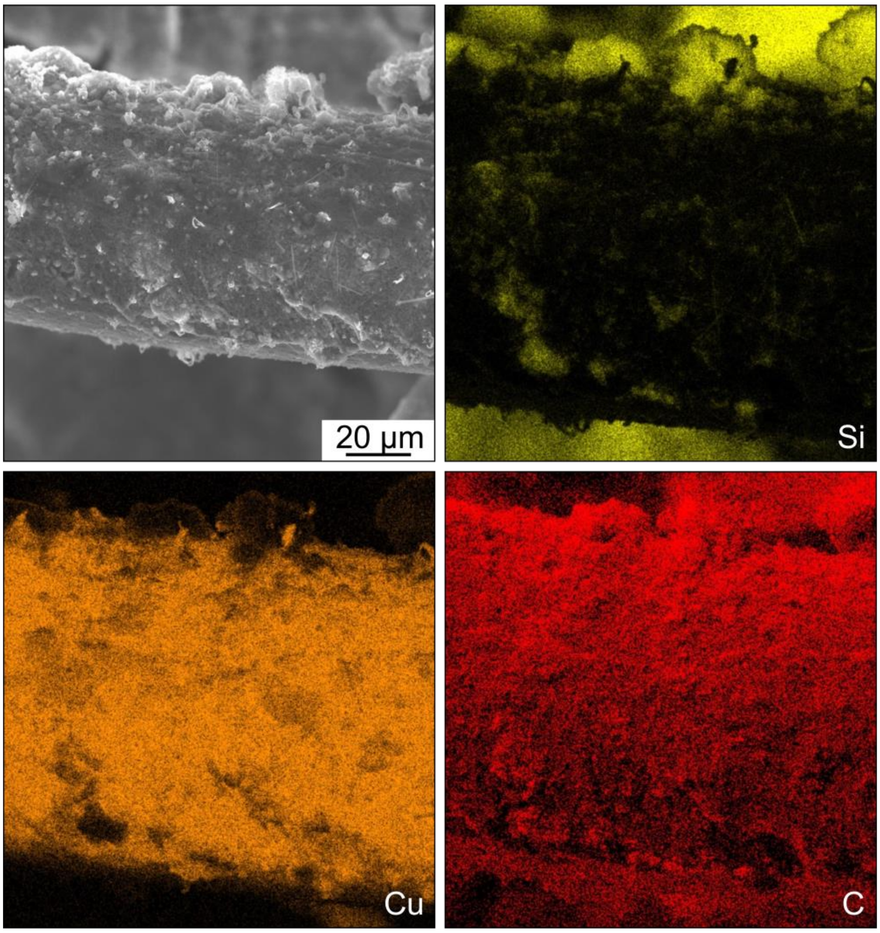

Figure S5. SEM image of a cycled Si-based electrode with 3D current collector and the corresponding EDX maps showing the distribution of Si, Cu and C. The electrode was somewhat cleaned (mechanically) prior to imaging to probe the contact area between the current collector and the electrode. According to EDX, the microwire surface is primarily covered by a layer of carbon black, thus indicating strong interaction (high affinity) between the copper and the carbon black/polymer binder composite. This "carbon shell" likely serves as the interface between the Cu microwires and the Si particles, yet the detailed relationship among Cu, C, polymer binder and Si needs further study.

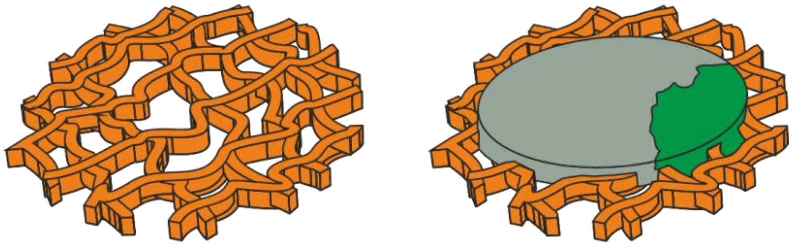

Figure S6. Schematic of Si-based electrodes with 3D current collector. (left) The same slurry used for the preparation of "conventional" electrodes was drop cast onto the embroidered current collector. (right) The slurry occupies the entire pore space between the Cu microwires. Because of shrinkage during the drying process, void

space (cracks etc.) develops between the electrode composite and the Cu wires. However, the different electrode regions (e.g., the one in green) remain electrically connected, while the void space likely helps to better accommodate the volume changes during electrochemical cycling.
